# Latent Structure of Combined Autistic and ADHD Symptoms in Clinical and General Population Samples: A Scoping Review

**DOI:** 10.3389/fpsyt.2021.654120

**Published:** 2021-12-20

**Authors:** Aneta D. Krakowski, Peter Szatmari, Jennifer Crosbie, Russell Schachar, Eric Duku, Stelios Georgiades, Evdokia Anagnostou

**Affiliations:** ^1^Department of Psychiatry, University of Toronto, Toronto, ON, Canada; ^2^Hospital for Sick Children, Toronto, ON, Canada; ^3^Centre for Addiction and Mental Health, Toronto, ON, Canada; ^4^Department of Psychiatry and Behavioural Neurosciences, McMaster University, Hamilton, ON, Canada; ^5^Offord Centre for Child Studies, McMaster Children's Hospital and McMaster University, Hamilton, ON, Canada; ^6^Bloorview Research Institute, Holland Bloorview Kids Rehabilitation Hospital, Toronto, ON, Canada; ^7^Department of Pediatrics, University of Toronto, Toronto, ON, Canada

**Keywords:** ASD, ADHD, scoping review, factor analysis, latent class analysis

## Abstract

**Background:** Many phenotypic studies have estimated the degree of comorbidity between Autism Spectrum Disorder (ASD) and Attention Deficit Hyperactivity Disorder (ADHD), but few have examined the latent, or unobserved, structure of combined ASD and ADHD symptoms. This is an important perquisite toward better understanding the overlap between ASD and ADHD.

**Methods:** We conducted a scoping review of studies that examined the factor or latent class structure of ASD and ADHD symptoms within the same clinical or general population sample.

**Results:** Eight studies met final inclusion criteria. Four factor analysis studies found that ASD and ADHD domains loaded separately and one found that some ASD and ADHD domains loaded together. In the three latent class studies, there were evidence of profiles with high levels of co-occurring ASD and ADHD symptoms.

**Conclusions:** Our scoping review provides some evidence of phenotypic overlap between ASD and ADHD at the latent, or unobserved, level, particularly when using a “person-centered” (latent class analysis) vs. a “variable-centered” (factor analysis) approach.

## Introduction

There has been increasing interest in the issue of comorbidity in Autism Spectrum Disorder (ASD). One of the most provocative issues in this area is the comorbidity with Attention Deficit Hyperactivity Disorder (ADHD). Comorbidity is defined as the association of two independent disorders occurring more often than expected by chance alone (estimated as the multiplicative product of the prevalence of each disorder alone) (1). In the Diagnostic and Statistical Manual of Mental Disorders (DSM) IV (2), it was not possible to make a diagnosis of ADHD in the presence of ASD as it was believed that ADHD symptoms were largely attributable to ASD and were considered part of its clinical presentation. This restriction has been removed in the DSM 5 (3) largely as a result of empirical studies estimating the degree of comorbidity between the disorders. A recent systematic review and meta-analysis found that 22% of children with ASD also met criteria for ADHD, with a higher estimate (34%) in clinical samples compared to community samples (4).

The mechanism for this comorbidity between ASD and ADHD is not clear, however. Possible explanations include: (1) measurement error (the way we measure the two disorders) (2) one disorder acting as a risk factor for the other, or (3) a common etiology leading to two different disorders [i.e., pleiotropy; that is a single genetic mechanism causing multiple phenotypes (5)]. Measurement error, or reporting bias, shared between two sets of symptoms or behaviors can be observed especially when the same informant reports on both disorders, and high scores on one disorder may bias an informant to rate an individual high (or low) on another disorder. This especially becomes a problem when diagnostic criteria overlap, as described by Caron and Rutter (1). In the DSM 5, there does not appear to be any overlap among ASD and ADHD diagnostic symptoms, therefore the comorbidity between ASD and ADHD cannot be directly attributed to overlap in diagnostic criteria alone although informant bias influencing ratings in other ways may still be a problem. A second possibility for comorbidity between ASD and ADHD is that one disorder may be a risk factor for the development of the second disorder. In this case it would be more likely that ASD is a risk factor for the development of ADHD as symptoms of ASD manifest slightly earlier in the course of a child's development than those of ADHD (6). However, a plausible causal chain linking the disorders is difficult to imagine let alone demonstrate empirically. A more likely possibility is that ASD and ADHD share some degree of common pathophysiology.

Reports on the genetics of ASD and ADHD suggest that the two disorders share several genetic risk factors (7–11). These include rare *de novo* copy number variants (12), such as those disrupting ASTN2 and TRIM33 (13). It is possible that at least in a sub-group of cases the same genetic risk factor(s) lead to the two disorders occurring in the same individual (i.e., pleiotropy). Shared neuroimaging endophenotypes between the two disorders has also been demonstrated when examining white matter indices (14, 15), functional networks (16, 17), and structural correlates (18, 19). Moreover, shared cognitive endophenotypes in executive functioning (20–22) and social cognition (23, 24) have been found between the two disorders.

Further evidence of pleiotropy might be investigated by an examination of the latent structure of both disorders. Factor analysis and latent class analysis are two complimentary approaches used to investigate latent structure (25). Factor analysis focuses on identifying phenotypic domains (it is “variable centered”), while latent class analysis focuses on identifying classes of individuals (it is “person centered”). Factor analytic techniques decompose a set of observed variables into a smaller group of unobserved latent constructs and could be useful in trying to decompose symptoms in ASD and ADHD into smaller more homogenous underlying phenotypic domains. Latent class analysis, on the other hand, helps derive homogenous subgroups in a heterogeneous sample of individuals (26) and could be useful in identifying subgroups of individuals with similar ASD and ADHD symptom profiles.

If shared risk factors were an explanation for the comorbidity between ASD and ADHD then it follows that ASD and ADHD symptoms might reveal a common latent construct, either on a latent factor and/or latent class level. For example, if factor analysis studies find that ASD and ADHD domains overlap at the latent phenotypic level in a subgroup of cases, the comorbidity between the two disorders might be partially explained. In addition, latent class analysis of ASD and ADHD symptoms in population-based or clinical samples might reveal classes with increased levels of ASD and ADHD symptoms or classes in which certain ASD and ADHD symptoms (i.e., social communication and hyperactivity/impulsivity) are increased, further providing a partial explanation for the comorbidity between ASD and ADHD.

There have been many factor analysis studies examining the factor structure of ASD and ADHD separately. An increasing amount of research has emerged showing that ASD is phenotypically composed of two domains; a social-communication domain and a restricted, repetitive, behaviors and interests (RRBI) domain (27, 28). This two-factor model is now incorporated in the DSM 5 which characterizes ASD as composed of impairments in social communication and a pattern of restricted, repetitive behaviors and interests. The majority of ADHD factor analysis studies report that symptoms of the disorder separate into two factors; inattention/distractibility and hyperactivity/impulsivity (29, 30). It is less clear what the factor structure would be when ASD and ADHD symptoms are analyzed together in the same participants.

Latent class studies in ASD have provided evidence for large variation in symptom severity and the possibility that different subgroups within ASD are associated with other variables such as cognitive abilities, level of functioning, and developmental trajectories (31–35). Using a combination of factor analysis and latent class analysis (factor mixture modeling), Georgiades et al. (31) reported that ASD consists of three different homogenous subgroups with different levels of symptom severity in the restricted repetitive behaviors and interests (RRBI) and social communicative domains. Heterogeneity is also evident in ADHD, and latent class studies have provided support for the existence of different subgroups based on symptom severity in the inattentive and hyperactivity/impulsivity domains (36–38). Latent class analysis of combined ASD and ADHD symptoms can shed further light on the heterogeneity of ASD and ADHD by identifying patterns of ASD and ADHD symptom overlap in clinical and general population samples.

The aim of the present review is to synthesize the existing published literature examining latent domains and latent classes of combined ASD and ADHD symptoms in clinical and general population samples. We chose to do a scoping review instead of a systematic review as we are interested in assessing the literature more broadly in terms of methodology and samples and cannot assess for methodological quality, as there are no critical appraisal guidelines that we are aware of for factor analysis or latent class studies.

## Methods

The Preferred Reporting Items for Systematic Reviews and Meta-analyses (PRISMA) guidelines for Scoping Reviews (39) and the framework outlined by Arksey and O'Malley (40) were used to structure our review. A literature search was conducted in three databases (Medline, PsychINFO, EMBASE) the third week of May 2020 with no restrictions on year of publication or publication type. A combination of MeSH-terms/index words and free text were used to search the databases. A medical librarian was also used to help inform the search methodology. Search terms related to ASD, ADHD, factor analysis, and latent class analysis. For example, the search in PsychINFO appears as follows: (exp Autism Spectrum Disorders OR asd^*^ OR autis^*^ OR asperger^*^) AND (exp Attention Deficit Disorder OR exp Attention Deficit Disorder with Hyperactivity OR adhd) AND [(exp Factor Analysis OR exp Principal Component Analysis OR exp Latent Class Analysis OR exp Latent Profile Analysis OR exp Cluster Analysis OR exp Latent Variables OR (factor^*^ adj8 analy^*^) OR (principal^*^ adj8 component^*^) OR (factor^*^ adj8 structure^*^) OR (measurement^*^ adj8 structure^*^) OR (mixture^*^ adj8 model^*^) OR (latent^*^adj8 class^*^) OR (latent^*^adj8 profile^*^) OR cluster^*^].

**Figure 1:**
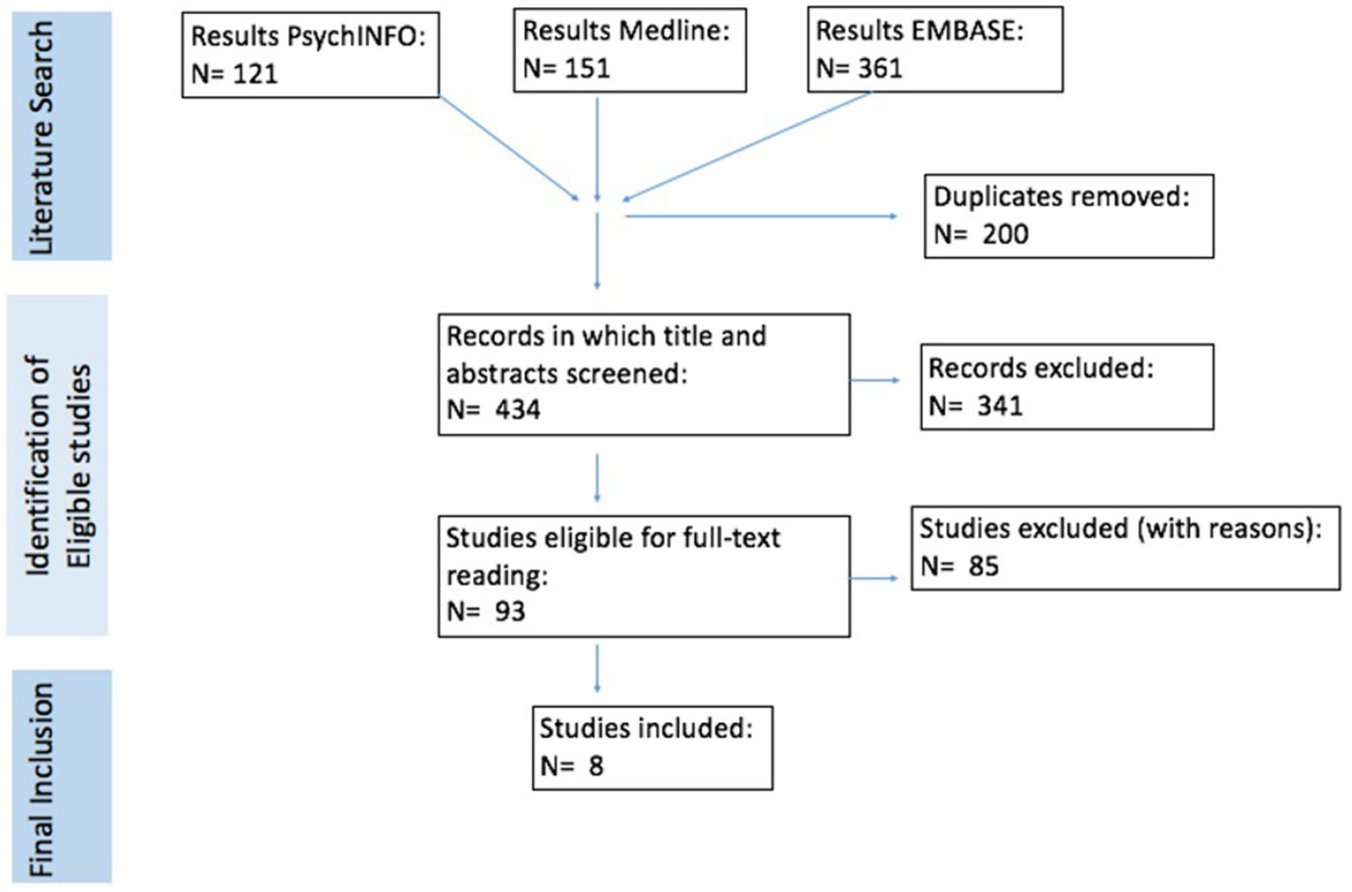
Summary of the search and selection process.

A summary of the search and selection process is outlined in Figure 1. AK screened titles and abstracts for eligibility and manually removed duplicates. Two of the authors (AK and PS) read the studies that were determined to be eligible for full-text reading. There were no differences in studies determined to be eligible for inclusion between AK and PS. A study was eligible for inclusion if the following criteria were met: (a) the study used a clinical sample of individuals with ASD or ADHD, or a sample of individuals from the general population, (b) used measurement tools that addressed symptoms of both ASD and ADHD, (c) used factor analytic techniques to examine the structure of combined ASD and ADHD symptoms OR used latent class analysis or clustering techniques to identify subgroups of individuals based on combined ASD and ADHD symptoms, (d) was published in English. We included all studies that used clinical samples with ASD or ADHD, regardless of the DSM version used to diagnose the clinical sample (i.e., DSM-IV, DSM-III-TR). This included clinical samples diagnosed under the umbrella of autism spectrum disorders in previous DSM versions (autistic disorder, Asperger syndrome and pervasive developmental disorder-not otherwise specified). We excluded studies in which we could not retrieve the full article and studies not published in peer reviewed journals.

In the studies that met inclusion criteria, AK extracted the data. The following descriptive data was extracted: sample size, sampling frame, demographic variables (gender, age, IQ), and measurement tools used to assess ASD and ADHD symptoms. Descriptive data for the study by Ghanizadeh (41) was extracted from two studies published previously which used the same study population and characterized the demographic variables and measurement tools used to assess ASD and ADHD symptoms (42, 43). For the study by Ronald et al. (44), sample size and demographic variables were extracted from the group used in the principal component analysis since the study used a different number of participants for the genetic analysis. In each factor analysis study, the following was extracted: the factor analysis technique (i.e., EFA vs. PCA) used, the rotation method used, the factor solutions, and the amount of variance explained by the factor model. In each latent class study, the following was extracted: the analysis technique used, the number/type of latent classes, and the proportion of participants compromising each class.

## Results

### Search Results

A summary of the search results is outlined in Figure 1. Of the 434 records identified in the search strategy, 93 unique studies were included for full-text reading. Eighty-five of these studies were excluded. Eight studies did not meet inclusion criteria as they did not use factor analytic or clustering techniques but instead examined the relationship between ASD and ADHD using other statistical methods. Twenty studies used factor analytic or clustering techniques, but the variables examined were not specific to ASD or ADHD symptoms and were therefore excluded. As other symptoms confound the analysis, they are less useful in determining the factor structure of combined ASD and ADHD symptoms specifically. One factor analytic study did not examine the factor structure of combined ASD and ADHD symptoms. Eighteen studies performed factor analysis or clustering only on ADHD symptoms or ASD symptoms. Thirteen studies did not do factor analysis or clustering on ASD/ADHD symptoms, but on other variables, i.e., neuropsychological test scores, demographic characteristics. Twenty studies did not have the full article available or were not published in a peer review journal. Finally, one study used latent classes from one of the included studies for further analysis. Eight studies met final inclusion criteria, five of these were factor analysis studies and three were latent class studies.

### Factor Analysis Studies

Characteristics and results of the included factor analysis studies are outlined in Table 1. Among the five studies that met final inclusion criteria, three used clinical samples. Ghanizadeh (41) used a clinical sample of children with ASD, Martin et al. (46) used a clinical sample of children with ADHD, and Krakowski et al. (47) used a clinical sample of children with ASD or ADHD. Two studies used children from the general population (41, 44). The sample sizes ranged from 65 to 7, 868.

**Table 1:** Characteristics of the included factor analysis studies.

**References**	**Sampling frame**	**Analysis**	**Sample age**	**IQ**	**Questionnaires used**	**Factors/components**
Ghanizadeh et al. (41) Journal IF: 4.785^*^	General Population Iran *n* = 1,600 M:800, F: 800	EFA	Mean (SD): 9.1 (1.4) yrs	Unknown; students with special education needs excluded	ASD: 12 CSI-4 items ADHD: 18 CSI-4 items	1. ADHD (inattentive symptoms) 2. ADHD (hyperactive symptoms) 3. ADHD (impulsivity symptoms) 4. ASD 5. ASD 6. ASD
Ghanizadeh et al. (45) Journal IF: 1.852^*^	Clinical sample (ASD) Iran *n* = 65 M: 49, F: 16	EFA	Mean (SD): 7.3 (2.4) yrs	Unknown; intelligence level was not an exclusion criteria	ASD: 12 ADI-R items ADHD: 18 K-SADS items	1. ASD 2. ADHD
Ronald et al., (44) Journal IF: 6.673^*^	Population sample (CATSS) Sweden *n* = 7,868 M: 3,956, F: 3,912	PCA	9 and 12 yrs	Unknown	ASD: 13 A-TAC items ADHD: 18 A-TAC items	1. ASD (social impairment) 2. ASD (communication impairment) 3. ASD (RRBIs) 4. ADHD (inattention) 5. ADHD (hyperactivity) 6. ADHD (impulsivity)
Martin et al., (46) Journal IF: 4.291^*^	Clinical sample (ADHD) UK *n* = 560 M:473, F: 87	EFA	Mean (SD): 10.4 (3.0) yrs	Mean (SD): 84.6 (13.9)	ASD: 39 SCQ items ADHD: 18 CAPA items	1. ADHD (Inattentive) 2. ASD (Social factor) 3. ASD/ADHD (Rigidity/hyperactivity)
Krakowski et al., (47) Journal IF: 7.509^*^	Clinical sample (ASD or ADHD) (POND) Canada ASD *n* = 303 M: 242, F: 61 ADHD *n* = 319 M: 253, F: 66	PCA	ASD Mean (SD): 11.2 (3.4) ADHD Mean (SD): 10.1 (2.7)	ASD Mean (SD): 87.5 (24.6) ADHD Mean (SD): 97.5 (15.9)	ASD: 39 SCQ items ADHD: 18 SWAN items	1. ADHD (Inattentive) 2. ADHD (Hyperactivity/impulsivity) 3. ASD (RRBI) 4. ASD (Social communication)

*ADHD, Attention Deficit Hyperactivity Disorder; ADI-R, Autism Diagnostic Interview- Revised; ASD, Autism Spectrum Disorder; A-TAC, The Autism-Tics, AD/HD and other Comorbidities Inventory; CAPA, The Child and Adolescent Psychiatric Assessment; CSI-4, Child Symptom Inventory 4; CATTS, Child and Adolescent Twin Study in Sweden; EFA, Exploratory Factor Analysis; K-SADS, Kiddie Schedule for Affective Disorders and Schizophrenia; IF, Impact Factor; PCA, Principal Component Analysis; yr(s), year(s); POND, Province of Ontario Neurodevelopmental Disorder Network Database; SCQ, Social Communication Questionnaire; TD, Typically Developing*.

* *The journal impact factor is based off 2020 metrics (48)*.

The mean age of the participants in the studies ranged from 7 to 12 years of age. While Ghanizadeh (41) excluded children with special education needs, intelligence level was not an exclusion criterion in Ghanizadeh (45). Amongst the two studies that reported on IQ, the mean was 87.5 for children with ASD (47), and the mean was 84.6 (46) and 97.5 (47) for children with ADHD. Krakowski et al. (47) measured IQ using age-appropriate Weschler or Stanford-Binet scales, and Martin et al. (46) measured IQ using Weschler scales.

Although the measurement tools used for ASD symptoms varied among the five studies, they all used informant-based reports and individual items were used for the factor analyses. All tools also incorporated a mix of items from the DSM-5 social communication and RRBI domains. Martin et al. (46) and Krakowski et al. (47) used the Social Communication Questionnaire (SCQ), a 40-item questionnaire developed by Berument et al. (49) that is based on the Autism Diagnostic Interview (50). The SCQ asks parents or caregivers to indicate the presence or absence of certain behaviors in order to help screen for autism. The SCQ has good internal consistency reliability with a Cronbach's α of 0.90 [a score above 0.70 is considered acceptable (51)] and good concurrent criterion validity, with a sensitivity of 0.85 and a specificity of 0.75 for differentiating ASD from other diagnoses (52). Ghanizadeh (45) used 12 ASD items based off DSM-IV criteria. Ronald et al. (44) used 13 ASD items obtained from the Autism-Tics, AD/HD, and other Comorbidities (A-TAC) inventory, which is a telephone interview. The A-TAC has good inter-rater reliability with an intraclass correlation coefficient (ICC) of 1.00 for ASD [above 0.70 is considered acceptable (51)], and good concurrent criterion validity, with a sensitivity of 0.89, specificity of 0.78, and an area under the curve (AUC) of 0.88 for ASD (53) [an AUC above 0.70 is considered acceptable (54)]. Ghanizadeh (41) used 12 ASD items from the Child Symptom Inventory (CSI)-4 (55, 56) which has good internal consistency reliability with a Cronbach's α of 0.73 for the autism subscale (57) and good concurrent criterion validity for autism with a sensitivity of 0.97, specificity of 0.93, and AUC of 0.99 using parent ratings from a public school sample (58). Although there were some differences in the measurement tools used for ADHD symptoms among the four studies, they were all informant-based reports and used 18 ADHD items with similar wording. Krakowski et al. (47) used the Strengths and Weaknesses of ADHD Symptoms and Normal Behavior Rating Scale (SWAN) (59), an 18-item questionnaire based on DSM-IV ADHD criteria. The SWAN has good internal consistency reliability with a Cronbach's α of 0.88 and good concurrent criterion validity with a sensitivity of 0.58 and specificity of 0.98 (60).The other four studies used ADHD items from general screening questionnaires for childhood psychiatric disorders or broad interviews for childhood psychiatric disorders.

Ghanizadeh (45) used ADHD items from the Kiddie Schedule for Affective Disorders and Schizophrenia (K-SADS) Farsi version, which has good inter-rater reliability with a kappa of 0.69 for ADHD, and good concurrent criterion validity with a sensitivity of 1.00 and specificity of 0.89 for ADHD (61). Ghanizadeh (41) used ADHD items from the CSI-4, which has good internal consistency reliability with a Cronbach's α of 0.91 for ADHD and good concurrent criterion validity with a sensitivity of 0.80 and specificity of 0.58 for ADHD (57). Ronald et al. (44) used ADHD items from the A-TAC, which has good inter-rater reliability with an ICC of 1.00 for ADHD, and good concurrent criterion validity, with a sensitivity of 0.92, specificity of 0.75, and an AUC of 0.91 for ADHD (53). Martin et al. (46) used ADHD items from the Child and Adolescent Psychiatric Assessment (CAPA) (62), which has good reliability and validity for various psychiatric disorders (62, 63); however, specific values for demonstrating the reliability and validity for ADHD could not be found in the literature.

In terms of the analysis used in the studies, three studies used exploratory factor analytic (EFA) techniques (41, 45, 46) and two used principal component analysis (PCA) (44, 47). Both studies on the general population (41, 44) used a varimax rotation in their analysis, not allowing ASD and ADHD factors to correlate. In contrast, the studies on the clinical population samples that reported on rotation method used oblique rotations in their analysis (46, 47), allowing factors to correlate.

In four out of the five studies, ASD and ADHD symptoms loaded separately on different factors. Ronald et al. (44) and Ghanizadeh (41) both used general populations and found six factor solutions composed of three ASD factors and three ADHD factors accounting for 56 and 53 % of the variance, respectively. Ghanizadeh (41) used a clinical sample of children with ASD and found a two-factor solution composed of one ASD factor and one ADHD factor accounting for 37 % of the variance. Krakowski et al. (47) used a clinical sample of children with ASD or ADHD and found a four-factor solution composed of two ASD factors and two ADHD factors accounting for 55% of the variance. In contrast to the other four studies, Martin et al. (46) used a sample of children with ADHD and found a three factor solution composed of a social factor, inattentiveness factor, and a third factor in which rigidity symptoms and hyperactive-impulsive symptoms grouped together. This solution accounted for 35% of the variance.

### Latent Class Studies

Characteristics and results of the included latent class analysis studies are outlined in Table 2. Among the three studies that met inclusion criterion, two used children from the general population (65, 66) and one used a mix of children from the general population and a clinical sample of children with ASD, ADHD, ASD +ADHD, and their non-affected siblings (64). The sample sizes ranged from 378 to 5,383. The sample age among the studies ranged from 5 to 17 years of age. All three studies reported that their participants IQ was above 70, the former cut-off for intellectual disability in the DSM-IV (67). IQ was measured in the three studies using age appropriate Weschler scales.

**Table 2:** Characteristics of the included latent class analysis studies.

**References**	**Sampling frame**	**Analysis**	**Sample age**	**IQ**	**Questionnaires used**	**Classes**
van der Meer et al. (64) Journal IF: 8.829^*^	General population and clinical sample (ASD, ADHD, or ASD + ADHD) (SPIDER, BOA) Netherlands *n* = 644	LCA	5-17 yrs	At least 70	ASD: 3 SCQ subscales ADHD: 5 CPRS-R:L subscale scores	1: “Normal” class (9.0 %) 2: “Normal” class (9.2 %) 3: “ADHD” class (16.9%) 4: “ADHD(+ASD)” class (23.2 %) 5: “ASD(+ADHD)” class (41.6 %)
Van der Meer et al. (65) Journal IF: 3.256^*^	General population (SPIDER) Netherlands *n* = 378	LCA	6-13 yrs M:F 1:1	At least 70	ASD: 5 AQ subscales ADHD: 2 SWAN subscales	1: Low ASD, Low ADHD (10.1%) 2: Medium ASD, Medium ADHD (54.2%) 3: High ASD, High ADHD (13.2%) 4: ADHD > ASD (18.3%) 5: ASD > ADHD (4.2%)
St. Pourcain et al. (66) Journal IF: 8.829^*^	General Population (AVON) England *N* = 5,383	LCGA	4-17 yrs	At least 70	ASD: 12 SCD items ADHD: 5 SDQ items	*ASD:* 1. Persistently Impaired: 10% 2. Low-risk: 90 % *ADHD* 1. Persistently Impaired: 3.94% 2. Intermediate risk: 8.07% 3. Childhood-Limited (5.25%) 4. Low-risk (82.74%)

*AQ, Autism Quotient; AVON, Avon Longitudinal Study of Parents and Children; BOA, Biological Origins of Autism; CPRS-R:L, Connors Parent Rating Scale Revised Long; IF, Impact Factor; LCA, Latent Class Analysis; LCGA, Latent Class Growth Analysis; SCD, Social Communication Disorder Questionnaire; SDQ, Strengths and Difficulties Questionnaire; SCQ, Social Communication Questionnaire; SPIDER, Schoolkids Project Interrrelating DNA and Endophenotype Research; SWAN, Strengths and Weakness of ADHD Symptoms and Normal Behaviour Rating Scale; yr(s), year(s)*.

* *The journal impact factor is based off 2020 metrics (48)*.

All three studies used informant-based reports for ASD and ADHD symptoms. While two of the studies used ASD and ADHD subscale scores (64, 65), one of the studies used ASD and ADHD item scores (66).

All studies used questionnaires specific to ASD to measure ASD symptoms. Pourcain et al. (66) used the Social Communication Disorder Questionnaire (SCD), which has good internal consistent reliability with a Cronbach's α of 0.93 (68) and good concurrent criterion validity for autism with a sensitivity of 0.90, specificity of 0.75 and AUC of 0.93 when using a general population-based sample, (69). Van der Meer et al. (64) used the Autism Quotient (AQ) which has good internal consistency reliability with Cronbach's α ranging from 0.63 to 0.77 in the five domains of the AQ (70), and good concurrent criterion validity with a sensitivity of 0.79 and specificity of 0.98 (when using a cut off of 32) (71). Van der Meer et al. (65) used the Social Communication Questionnaire (SCQ), which has good reliability and validity as described above.

Two studies used questionnaires specific to ADHD (65) or ADHD and related symptoms (64). Van der Meer et al. (65) used the SWAN, which has good reliability and validity as described above. Van der Meer et al. (64) used the Conner's Parent Rating Scale—Revised: Long version (CPRS-R:L) which has good internal consistency reliabilities with Cronbach's α ranging from 0.75 to 0.94 depending on the sex and age of the participants (72). The scale also has good concurrent criterion validity, with a sensitivity of 0.92 and specificity of 0.95 for ADHD (72). The study by Pourcain et al. (66) used the ADHD items from the Strength and Difficulties Questionnaire (SDQ), a general screening questionnaires for childhood psychiatric disorders. Stone et al. (73) evaluated parent scores across studies on the SDQ inattentive/hyperactivity domain and found good internal consistency reliability with an average Cronbach's α of 0.76, and good inter-rater reliability with an average correlation of 0.47. Goodman (74) found good concurrent criterion validity for the SDQ, with a sensitivity of 0.74 and specificity of 0.92 for ADHD using parent ratings.

In terms of the analysis used in the studies, van der Meer et al. (64, 65) used latent class analysis on ASD and ADHD symptoms together. The study by Pourcain et al. (66) used latent class growth analysis of social-communication and inattentive-hyperactive trajectories in parallel using four time points for social-communication symptoms (8, 11, 14, and 17 years of age) and seven time points for inattentive-hyperactive symptoms (4, 7, 8, 10, 12, 13, and 17 years of age).The authors did not examine joint trajectories. The two latent class studies found a five-class solution (64, 65), and the latent class growth analysis study (66) found two social-communication trait trajectories and four inattentive-hyperactive trait trajectories.

Van der Meer el al. (64) found two classes (9.0 and 9.2%) with similar levels of ASD and ADHD scores, both with low levels of ASD and ADHD scores that they labeled “normal classes.” Van der Meer et al. (65) found three classes with similar levels of ASD and ADHD scores; one with low ASD and low ADHD scores (10.1%), a second with medium levels of ASD and ADHD scores (54.2%), and a third with high levels of ASD and ADHD scores (13.2%). In the class with high levels of ASD and ADHD, the mean total AQ and SWAN scores were significantly higher than those of the low and medium classes; however, only 30% of children scored above the clinical cut-off on both measures. Both van der Meer et al. (64, 65) found one class (41.6, 4.2%, respectively) in which ASD scores were higher than ADHD scores, and one class (23.2%, 18.3%, respectively) in which scores were ADHD scores were higher than ASD scores. In the van der Meer et al. (65) study about 30% of the participants had ASD scores above the clinical cut-off in the ASD > ADHD group. Participants in the ADHD > ASD group were noted to have “intermediate” ADHD scores and it is unclear how many participants had ADHD scores above clinical cut-off. In the van der Meer et al. (64) study, participants in the ASD > ADHD group had ASD scores substantially above clinical cut-off, ADHD hyperactive/impulsive scores slightly above clinical cut-off, and ADHD inattentive scores below clinical cut-off. In the ADHD > ASD group, ADHD scores were substantially above clinical cut-off and ASD scores were slightly above clinical cut-off. While van der Meer et al. (64) found one class with only elevated ADHD scores, which were above clinical cut-off, van der Meer et al. (65) did not find such a class. Neither study found a class with only elevated ASD scores.

Finally, Pourcain et al. (66) found two social-communication trait trajectories: a persistently impaired group (10%) with an increased probability of expressing social-communication difficulties and a low risk group (90%). There were four inattentive-hyperactive trait trajectories: a persistently impaired group with a high probability of expressing inattentive-hyperactive symptoms (3.94%), an intermediate group with an intermediate probability of expressing inattentive-hyperactive symptoms (8.07%), a childhood limited group (5.25 %), and a low risk group (82.74%). Children in the persistently impaired social-communication group were mostly either part of the persistently impaired (32.3%) or intermediate (39.0%) inattentive-hyperactive groups. Children in the persistently impaired inattentive-hyperactive group were almost all part of the persistently impaired social-communication group (82.0%).

## Discussion

In four out of the five factor analysis studies included in our scoping review, ASD and ADHD domains loaded separately on different factors. Two of these studies were on population groups (41, 44) and two were on clinical samples, one on children with ASD (45) and one on children with ASD or ADHD (47). In contrast, in the clinical sample of ADHD children (46), some ASD and ADHD items were so highly correlated that they loaded together and reflected at least one common underlying developmental domain. In summary, most of the factor analysis studies identified in our scoping review suggest that ASD and ADHD symptoms have separate latent factors.

The three latent class studies included in our scoping review found evidence of latent classes with elevated levels of co-occurring ASD and ADHD symptoms meeting clinical thresholds. Two studies, both by van der Meer et al. (64, 65) (one from the general population and one from a combined ASD/ADHD clinical and general population sample), found five classes with differing levels of combinations of ASD and ADHD symptoms. In both studies, it was much more common for individuals to belong to a class characterized by ADHD>ASD than ASD>ADHD. The third latent class study, by Pourcain et al. (66), used a longitudinal general population cohort and by applying latent class growth analysis, found two ASD trajectories and four ADHD trajectories. Individuals in the high ASD trait trajectory class were also in the high ADHD trait trajectory class, but not vice versa. In summary, all the latent class studies from our scoping review suggest that specific latent subgroups with co-occurring ASD and ADHD symptoms exist, irrespective of DSM diagnosis.

An emerging theme from our scoping review is that ASD and ADHD show phenotypic overlap, with greater overlap seen on a “person” than a “variable” level. As four of the five factor analysis studies showed that ASD and ADHD symptoms loaded onto separate latent domains, it may be less likely that a shared latent ASD/ADHD construct can explain ASD and ADHD covariation. On the other hand, all the three latent class studies found that latent subgroups with high levels of co-occurring ASD and ADHD symptoms exist, providing a partial explanation for the comorbidity between ASD and ADHD and supporting the idea that ASD and ADHD share some degree of common pathophysiology.

Using a latent growth class analysis, Pourcain et al. (66) provide support for the longitudinal overlap between social-communication and ADHD trajectories among individuals in the general population, with those individuals in the high social communication trait trajectory class more likely to also be in the trajectory class with high ADHD traits. Using the same cohort, Stergiakouli et al. (9) found evidence of shared genetic etiology between the social-communication domain and ADHD. Overlap between social communication traits and ADHD symptoms is further supported by Pinto et al. (75) on both a phenotypic and genetic level using a population based twin sample.

Although Krakowski et al. (47) found that ASD and ADHD items loaded onto separate domains using a clinical sample (the POND cohort), a further profile analysis in the study comparing ASD and ADHD component scores found that there was no difference in hyperactive and inattentive component scores between the two diagnostic groups. This provides support for phenotypic overlap between the two disorders on a “person level.” Cross-disorder overlap using phenotypic data has also been found in the same cohort used by Krakowski et al. (47) (POND) in two recent studies. Kushki et al. (76) used a clustering approach on core phenotypic features and cortical thickness in children with ASD, ADHD and OCD and found that their derived clusters did not map well onto diagnostic categories. Similarly, Jacobs et al. (77) used a spectral clustering function approach on cortical thickness, subcortical volume, white matter fractional anisotropy, and behavioral measures (including core phenotypic features) in children with ASD, ADHD and OCD, and found that their derived clusters cut across diagnostic categories.

Interestingly, both latent class studies by van der Meer et al. (64, 65) (one from the general population and one from a combined ASD/ADHD clinical and general population sample), found the existence of a group with high scores on only ADHD symptomatology and found that the existence of a group with high scores on only ASD symptomatology was either non-existent or was very small. The authors discuss how this supports their theory that ASD and ADHD are part of a gradient overarching disorder hypothesis (21), in which ADHD and ASD are a single disorder. They also hypothesize that ADHD is on the milder end of this gradient, and support their theory by showing that the ADHD class is less impaired in cognitive functioning than the comorbid ASD/ADHD classes (64).

Although our scoping review only found one factor analysis study in which ASD and ADHD items loaded together (46) this finding is worthy of discussion. In their study, Martin et al. (46) specifically found that rigidity and hyperactivity/impulsivity items loaded together. The association between rigidity in ASD and hyperactivity-impulsivity in ADHD is supported to some extent in the literature. A recent familial study by Sokolova et al. (78) showed a direct association between RRBIs in autism and hyperactivity in ADHD using causal modeling. Moderate genetic correlations have also been shown between rigidity and hyperactivity-impulsivity in adult twin populations by Polderman et al. (79) (r = 0.64) and Ghirardi et al. (8) (r = 0.56). This supports the existence of a possible sub-group of ASD and ADHD in which ASD and ADHD may constitute one disorder driven by the hyperactive-impulsive domain and the RRBI domain loading together onto a common developmental domain. On the other hand, this group may be an artifact of the measurement tools used for factor analysis,

Various limitations arise from the sampling and methodology used in the eight studies included in the literature review. These make it difficult to make generalizations. First, there is variability in IQ among the study participants in the five factor analysis studies. Martin et al. (46) and Ghanizadeh (45) did not exclude children based on IQ, while Ghanizadeh (41) excluded children with special education needs. Although Krakowski et al. (47) did not exclude based on IQ, the authors did remove non-verbal children from their analysis which might have resulted in a higher mean IQ. Interestingly, the mean IQ of the ADHD population in the Martin et al. (46) study was below average at 84.6, which was likely due to 12% of the participants having an intellectual disability. Van der Meer et al. (64, 65) and Pourcain et al. (66) all included children with an IQ of at least 70. IQ is important to consider as IQ has been shown to have a negative linear relationship with ADHD symptoms (80–82). A similar negative correlation has been found between IQ and scores on an autism rating scale in children with pervasive developmental disorders (83). Overall, it is unclear how much variation in IQ may have influenced ASD and ADHD symptoms and therefore subsequent factor loadings and latent class groupings in the different studies.

Second, there is variability in the questionnaires used for ASD and ADHD items. While all included studies used ASD and ADHD items based on DSM-IV or DSM-5 criteria, they used different questionnaires and therefore the wording of the items differed between studies. Many were also general screening questionnaires for childhood psychiatric disorders (CSI-4, A-TAC, SDQ) or broad interviews for childhood psychiatric disorders (K-SADS, CAPA) and not specific to ASD or ADHD. This may have affected the robustness of the information obtained for the factor or latent class analysis. The studies by Martin et al. (46) and Krakowski et al. (47) used items from the SCQ in their analysis which contains a greater number of items compared to the other ASD questionnaires.

Third, all four studies included in the literature review used parent-based reports for the assessment of symptoms. ASD and ADHD symptoms may be difficult to distinguish by parents and may be interpreted differently between parents, teachers and clinicians. This is highlighted in a recent study by Grzadzinski et al. (84) which showed that ASD symptoms (especially social-communication symptoms) are interpreted differently by parents (using the ADI-R) and clinicians (using the Autism Diagnostic Observation Schedule) in children with ADHD. This suggests that clinician assessment of ASD and ADHD symptoms could be an important complement to parent questionnaires when exploring latent structure.

Fourth, the included studies had some variability in methodology, particularly the factor analysis studies. Two of the studies (41, 44) used varimax rotations which assumes there is independence between ASD and ADHD and does not allow the factors to correlate. By asking for distinct mutually exclusive constructs in their analysis, these studies would not have seen overlap (if it exists). This is in contrast to the study by Martin et al. (46) and Krakowski et al. (47) which used an oblique rotation and allowed ASD and ADHD factors to correlate.

Last, two of the latent class studies (64, 65) shared a cohort for their analysis therefore it is unclear if their findings are sample specific. The remaining latent class study used latent class growth analysis (66), which groups individuals based on symptom growth trajectories instead of symptoms at a fixed point in time, and therefore cannot directly be compared to other two latent class studies.

Should future studies that perform a combined factor analysis of ASD and ADHD items in clinical samples emerge, it is likely they will use clinical samples diagnosed using DSM-5 criteria. Most studies in our review used DSM-IV criteria for diagnosing children with ASD and ADHD, with the exception of Krakowski et al. (47), which used a combination of children diagnosed using DSM-IV-TR and DSM-5 criteria. This is an important consideration for future systematic reviews that may aim to compare clinical samples diagnosed using different DSM versions.

### Future Directions

Among the factor analysis and latent class studies included in our scoping review, there is variability in terms of sampling, measurement tools, analytic techniques, and on results. This emphasizes the ongoing need to investigate the latent domains and latent classes of combined ASD and ADHD symptoms as this is fundamental to understanding the high rates of comorbidity between ADHD and ASD in clinical and general population samples. Our review also highlights the importance of future studies clearly outlining the characteristics of the groups used (i.e., IQ) and the statistical methodologies employed so that meaningful comparisons can be made.

As there is variation in the samples used for factor analysis studies it is unclear whether demographic variables or diagnosis influence factor structure. By testing measurement invariance, it is possible to determine whether a factor structure is stable across subgroup membership defined by such variables (85). Since heterogeneity in ASD and ADHD is influenced by age (86–88), sex (89–91), and adaptive functioning (92–94), these are important variables to consider when examining measurement invariance. It would also be helpful to determine whether the underlying factor structure of ASD and ADHD symptoms is stable across time in the same group of individuals used in longitudinal studies.

Overall, our scoping review found that there is more evidence of phenotypic overlap at the “person” level than on a “variable” level. Future studies should aim to better characterize patterns of ASD/ADHD phenotypic overlap by using latent class techniques and should attempt to evaluate the clinical meaningfulness of these subgroups. Latent class growth analysis studies would be particularly useful in providing a longitudinal perspective regarding the pattern of ASD/ADHD overlap among subgroups of individuals. Since Pourcain et al. (66) included only social communication traits from ASD in their analysis, future latent class growth analysis studies of both social communication traits and restricted repetitive behaviors and interests (RRBIs) together with ADHD domains might provide a more comprehensive picture of ASD and ADHD trajectories. Furthermore, it would be helpful for future studies to examine joint ASD and ADHD trajectories in order to examine overlap in ASD and ADHD trajectories across time.

As studies have found evidence of ASD and ADHD domain-specific aetiological and phenotypic overlap (8, 44, 79, 95), for example between RRBIs and hyperactivity/impulsivity, it would helpful for future latent class studies to investigate whether evidence of transdiagnostic domain overlap exists within identified subgroups. This would be particularly important to investigate from a longitudinal perspective, given that some domains (inattention and social problems) have shown more persistent impairment than other domains (RRBIs and hyperactivity/impulsivity) (96).

Finally, given the complexity of the ASD/ADHD comorbid phenotype, future studies may also benefit from examining the latent structure of combined ASD and ADHD symptoms using factor analysis techniques combined with latent class analysis. A combined analysis of these two statistical techniques such as in factor mixture modeling (25, 31) could help determine whether the factor structure of combined ASD and ADHD symptoms varies among subgroups. For example, perhaps one ADHD sub-group has an overlapping factor structure with ASD in which hyperactive-impulsive traits load together with RRBIs.

### Strengths and Limitations of Scoping Review

As part of the scoping literature review, we used broad inclusive criteria to ensure all relevant studies using factor analytic techniques or clustering analysis were included. This also involved including both clinical and general population samples.

Although our literature review attempted to be comprehensive, limiting publication type to English and not searching through published abstracts may have excluded potentially relevant studies. Furthermore, while we had two reviewers read through the articles included for full-text reading, only one reviewer screened the titles and abstracts.

### Conclusions

We found eight studies in our scoping review (five factor analysis studies and three latent class studies). These studies suggest that while ASD and ADHD domains have separate/ different factor structure, there is a latent class of individuals with co-occurring ASD/ADHD symptoms. This provides some evidence of phenotypic overlap in ASD and ADHD clinical and population samples, particularly when using a “person-centered” vs. a “variable-centered” approach. Clinically, this suggests that symptoms from ASD and ADHD domains may be difficult to attribute to an isolated ASD or ADHD presentation, as they may actually represent an overlapping ASD and ADHD phenotype.

## Data Availability Statement

The original contributions presented in the study are included in the article/supplementary material, further inquiries can be directed to the corresponding author/s.

## Author Contributions

AK and PS conceived of the study. AK conducted the literature search, analyzed the data, and wrote the manuscript. PS assisted with the literature search. PS, JC, RS, ED, SG, and EA helped with the interpretation of results and contributed to editing of the manuscript. EA and PS supervised the research. All authors reviewed and approved the final revision of the manuscript.

## Funding

This research was conducted with the support of the Ontario Brain Institute (POND – PI:Anagnostou), an independent non-profit corporation, funded partially by the Ontario government.

## Author Disclaimer

The opinions, results and conclusions are those of the authors and no endorsement by the Ontario Brain Institute is intended or should be inferred.

## Conflict of Interest

PS has received royalties from Guilford Press and Simon & Schuster. RS is in on the Scientific advisory board for Highland Therapeutics and on the Scientific advisory board for ehave (psychological software company). RS also has equity in ehave. EA has received consultation fees from Roche and Takeda, royalties from APPI and Springer, and funding from SynapDx and Sanofi-Aventis. The remaining authors declare that the research was conducted in the absence of any commercial or financial relationships that could be construed as a potential conflict of interest.

## Publisher's Note

All claims expressed in this article are solely those of the authors and do not necessarily represent those of their affiliated organizations, or those of the publisher, the editors and the reviewers. Any product that may be evaluated in this article, or claim that may be made by its manufacturer, is not guaranteed or endorsed by the publisher.
